# Conserved metallomics in two insect families evolving separately for a hundred million years

**DOI:** 10.1007/s10534-014-9793-9

**Published:** 2014-10-09

**Authors:** Polychronis Rempoulakis, Negar Afshar, Beatriz Osorio, Martha Barajas-Aceves, Joanna Szular, Sohel Ahmad, Thilakasiri Dammalage, Ulysses Sto Tomas, Esther Nemny-Lavy, Mor Salomon, Marc J. B. Vreysen, David Nestel, Fanis Missirlis

**Affiliations:** 1 IAEA Laboratories, Insect Pest Control Laboratory, Joint FAO/IAEA Programme of Nuclear Techniques in Food and Agriculture, International Atomic Energy Agency, Seibersdorf, Austria; 2 Department of Entomology, Institute of Plant Protection, Agricultural Research Organization (ARO), The Volcani Center, Beit Dagan, Israel; 3 School of Biological and Chemical Sciences, Queen Mary University of London, Mile End Road, London, UK; 4 Departamento de Fisiología, Biofísica y Neurociencias, Centro de Investigación y de Estudios Avanzados del Instituto Politécnico Nacional, Av. IPN 2508, Zacatenco, Mexico City, Mexico; 5 Departamento de Biotecnología y Bioingenería, Centro de Investigación y de Estudios Avanzados del Instituto Politécnico Nacional, Av. IPN 2508, Zacatenco, Mexico City, Mexico; 6 Citrus Division, The Israel Cohen Institute for Biological Control, Plants Production and Marketing Board, Beit Dagan, Israel

**Keywords:** Physiology, Evolution, Genetics, Nutrition, Fruit flies of economic importance, Agriculture, Mediterranean fruit fly

## Abstract

Μetal cofactors are required for enzymatic catalysis and structural stability of many proteins. Physiological metal requirements underpin the evolution of cellular and systemic regulatory mechanisms for metal uptake, storage and excretion. Considering the role of metal biology in animal evolution, this paper asks whether metal content is conserved between different fruit flies. A similar metal homeostasis was previously observed in Drosophilidae flies cultivated on the same larval medium. Each species accumulated in the order of 200 µg iron and zinc and approximately ten-fold less manganese and copper per gram dry weight of the adult insect. In this paper, data on the metal content in fourteen species of Tephritidae, which are major agricultural pests worldwide, are presented. These fruit flies can be polyphagous (e.g., *Ceratitis capitata*) or strictly monophagous (e.g., *Bactrocera oleae*) or oligophagous (e.g., *Anastrepha grandis*) and were maintained in the laboratory on five distinct diets based on olive oil, carrot, wheat bran, zucchini and molasses, respectively. The data indicate that overall metal content and distribution between the Tephritidae and Drosophilidae species was similar. Reduced metal concentration was observed in *B. oleae*. Feeding the polyphagous *C. capitata* with the diet of *B. oleae* resulted in a significant quantitative reduction of all metals. Thus, dietary components affect metal content in some Tephritidae. Nevertheless, although the evidence suggests some fruit fly species evolved preferences in the use or storage of particular metals, no metal concentration varied in order of magnitude between these two families of Diptera that evolved independently for over 100 million years.

## Introduction

 An obvious consideration in the evolution of flies and all organisms is the physiologic use of metals as cofactors in proteins (Cyert and Philpott 2013; Godfrey and Glass 2011; Hansch and Mendel 2009). Metal homeostasis arises from the combination of regulating dietary absorption and organismal storage and excretion to ensure tissues and cells have sufficient metal ions available for biological use (Mandilaras et al. 2013; Southon et al. 2013; Tang and Zhou 2013). Dietary absorption, in turn, is under continuous environmental influence forming a key ecological factor defining each species’ niche (Forbes et al. 2009; Lang et al. 2012; Sharon et al. 2010). The extent to which specialized diets and natural selection affect the metal homeostasis systems in different insect orders has not been investigated.

Previous research has indicated that genetic change can influence metal homeostasis in *Drosophila melanogaster,* a commonly used fly in basic research. Mutations in the X-chromosome of *D. melanogaster* can cause dramatic changes in total body zinc accumulation (Afshar et al. 2013) and mutations in an iron transporter (Bettedi et al. 2011), or flies heterozygous for mutations in the iron storage protein ferritin (Gutierrez et al. 2013), accumulate less iron in their bodies. Similarly, flies heterozygous for mutations in *Syntaxin 5* accumulate less copper (Norgate et al. 2010). Changes in metal concentrations can also arise by RNA interference (Bahadorani et al. 2010; Soriano et al. 2013; Xiao et al. 2014). Furthermore, when nine species of Drosophilidae, chosen for their differences in ecology and behavior and because their full genomes had been available (Clark et al. 2007), were raised on the same larval medium, they showed a similar metal profile, suggesting that evolutionary mechanisms exist that shape, and conserve, metal homeostasis (Sadraie and Missirlis 2011). In this paper, we addressed the hypothesis that this relative stability of laboratory-maintained Drosophilidae metallomes is also conserved in other fruit flies.

Along with the family Drosophilidae, the family Tephritidae (true fruit flies) belongs to the insect order Diptera. The Tephritidae form a highly variable group of approximately five thousand described species belonging to five hundred genera (Aluja and Norrbom 1999; White and Elson-Harris 1994). The larvae in the majority of these species are phytophagous, feeding exclusively on plant tissues and pose a significant threat to agriculture in many regions of the world. The host range varies significantly among the different species, with some being highly polyphagous, e.g., the Mediterranean fruit fly *Ceratitis capitata* has 356 recorded fruit and vegetable hosts (Liquido et al. 1991; Hancock et al. 2000; De Meyer et al. 2002). Other species are strictly monophagous, e.g., the olive fruit fly *Bactrocera oleae*, which feeds only on the olive fruit mesocarp (Tzanakakis 2003), or oligophagous, feeding on a small range of hosts, e.g., the South American cucurbit fruit fly *Anastrepha grandis*. Colonies of fruit flies were maintained at the Insect Pest Control Laboratory (IPCL) of the joint Food and Agriculture Organization–International Atomic Energy Agency (FAO-IAEA) program of Nuclear Techniques in Food and Agriculture of the United Nations. Appropriate artificial larval diets provide all the necessary nutrients for the development of the flies. Rearing of the polyphagous and oligophagous flies on a common artificial medium is possible, but certain species have unique requirements and the use of natural hosts as rearing medium is still unavoidable. This is the case with species attacking Cucurbitaceae, like *A. grandis* (Gomes Silva and Malavasi 1993) and the African fruit fly *Dacus ciliatus* (Zur et al. 2009), which have both been maintained on their natural hosts (zucchinis) for a number of generations.

In this study, the metal contents in the bodies of newly emerged adults belonging to 14 different species of fruit fly, representing the major pests, were measured. With the exception of *D. ciliatus* and one population of *C. capitata* all other insects were maintained at the IPCL facilities. Whereas previous studies have compared species only within a single family representing perhaps 10–12 million years of separation, this resource was used to evaluate evolutionary mechanisms operating on insect metal homeostasis over a much longer period by comparing the families Drosophilidae and Tephritidae, which are estimated to have been divergent for 100 million years (Beverley and Wilson 1984; Wulbeck and Simpson 2000). In addition, a dietary variable (an olive oil supplement) was introduced experimentally to test its effect on metal accumulations and address the theory that the oil reduces tissue metal content. Only few previous studies have addressed whether olive oil might have metal chelating properties, and none of these involved tests with experimental animals (Briante et al. 2003; Paiva-Martins and Gordon 2005; Visioli and Galli 2002).

## Materials and methods

### Insects

In view of unfavorable climatic conditions in the area (Seibersdorf, Austria), fruit flies are not considered a quarantine pest in Austria and hence, the IPCL has a unique collection of fruit fly strains and species that allows for parallel and comparative studies of many different species. The origin and typical hosts of all strains used in this study are listed in Table 1. The number of generations the colonies were maintained at the IPCL and the history of each line are shown. Fruit flies representing four genera (*Bactrocera*, *Anastrepha*, *Dacus* and *Ceratitis*) were selected for analysis and experimentation. *Bactrocera* was represented with nine species, *Anastrepha* with three species, and *Ceratitis* and *Dacus* with one species each. The fly colonies were maintained in controlled bioclimatic rooms at a temperature of 25 ± 2 °C, humidity at 65 ± 5 %, and a photoperiod of light to dark cycles of 14:10 h. Eggs were collected daily from cages containing mature reproductive adults and they were placed on a thin paper towel on top of a thick layer of larval medium in 5 L trays. These trays were transferred to higher humidity (90 %) chambers where they were held until their pupation in vermiculite. Pupae were sieved and transferred to a dry room. After approximately 10 days the adults emerged in large Perspex cages (50 × 50 × 40 cm), with adult diet (sugar:yeast hydrolysed 3:1) and water provided ad libitum. Two to four days following emergence, a large number of flies (>500) from each species were collected in plastic vials, freeze dried using standard procedures and shipped for metal content analysis. *D. melanogaster* was used as a control.

**Table 1:** Characteristics and origin of species used in this study

Species and level of phagy	Natural host*	Origin of strain	Population	Generation
1-1 *Bactrocera oleae—m*	Olives	Italy	Wild type	5
1-2 *Bactrocera oleae*		Greece	Laboratory	>500
1-3 *Bactrocera oleae*		Israel	Lab-wild hybrid	100
2-1 *Bactrocera dorsalis—p*	Apples, guava, banana	Thailand	Wild type	20
2-2 *Bactrocera dorsalis*		China, Yunnan	Laboratory	6
2-3 *Bactrocera dorsalis*		Hawai	Lab GSS strain	65
3 *Bactrocera tryoni—p*	Apples, apricots, coffee	Australia	Laboratory	32
4 *Bactrocera cucurbitae—o*	Pumpkins	Mauritius	Wild type	19
5 *Bactrocera carambolae—p*	*A. carambola*, Syzigium, banana	Surinam, Paramaribo	Wild type	16
6 *Bactrocera papaya—p*	Banana, mango, papaya	Malaysia, Serdang	Wild type	12
7 *Bactrocera invadens—p*	Kashew nut, citrus, pine apple	Kenya	Laboratory	37
8 *Bactrocera zonata—o*	Peach, guava, mango	Mauritius	Laboratory	19
9 *Bactrocera philippinensis—p*	Breadfruit, Sizygium, mango	Philippines, Guimaras	Wild type	17
10* Anastrepha ludens—p*	*M. indica*, prunus, citrus	Mexico	Laboratory	25
11 *Anastrepha fraterculus—p*	*P. java*, prunus, citrus	Brazil, Vacaria	Wild type	22
12 *Anastrepha grandis—o*	Pumpkins	Brazil**	Wild type	3
13-1 *Ceratitis capitata—p*	~356 hosts	Argentina	Wild type	70
13-2 *Ceratitis capitata*		Austria	Lab GSS strain	8
13-3 *Ceratitis capitata*		Israel	Laboratory	~500
14 *Dacus ciliatus—o*	Pumpkins	South Israel	Laboratory	50
15 *Drosophila melanogaster—p*	Yeasts	Italy, Tannes	Wild type	>500

* Host data from (White and Elson-Harris 1994) and from the invasive species compendium cabi.org

** Collected from fruits imported from Brazil

*m* Monophagus, *o* oligophagus, *p* polyphagous, *GSS* genetic sexing strain

### Diets

The larval stage of the different fly species were reared in the following media.

Olive fly diet (i) (olive fly and Mediterranean fruit fly): a-cellulose 28 %, brewer’s yeast 7.6 %, soya hydrolyzed 3 %, sugar 2 %, olive oil 2 %, Tween 80 0.75 %, potassium sorbate 0.05 %, nipagin 0.2 %, HCl 0.45 %, Water 55 %.

Carrot diet (ii) (*Anastrepha* and *Bactrocera* species): carrot powder 15 %, brewer’s yeast 7 %, sodium benzoate 0.24 %, nipagin 0.19 %, HCl 0.8 %, water 77 %.

Mediterranean fruit fly diet (iii) (Mediterranean fruit fly): wheat bran 24.2 %, brewer’s yeast 8.1 %, sugar 16.2 %, sodium benzoate 0.5 %, citric acid 1.8 %, water 49.2 %. A variation of this diet was also used where indicated: wheat bran 26.8 %, brewer’s yeast 8.1 %, sugar 12.1 %, nipagin 0.4 %, HCl 1.6 %, water 51 %.

Zucchini diet (iv) (*A. grandis* and *D. ciliatus*): fresh, organically grown zucchinis.

*Drosophila* diet (v) (*D. melanogaster*): molasses 12.5 %, brewer’s yeast 10 %, agar 1.6 %, gelatin 0.3 %, propionic acid 1 %, water 74.6 %.

All ingredients were purchased from local suppliers and extra virgin olive oil was used where indicated.

### Atomic absorption spectrometry

Insects were freeze dried, weighed, and 200 µg were digested with nitric acid under heating conditions previously described (Sadraie and Missirlis 2011). Total Cu, Fe, Mn and Zn concentration in the insects were measured in the flame atomic absorption spectrometry (Avanta M System 300 GF 3000 S/N 10288). Prior to analysis the samples were filtered to remove impurities and any undigested material (Barajas-Aceves 2005).

### Diet manipulation and fly growth and reproduction

For the experiment of the Mediterranean fruit fly reared in its own and in olive fly diet with (2 %) and without (0 %) olive oil, a laboratory colony was used as source. Eggs were collected daily (~24 h old) and were transferred to the three different diets. Essential biological parameters (egg-pupa recovery, pupal weight, and adult emergence) were recorded following the (FAO/IAEA/USDA 2003). The number of eggs placed on the diet in trays was counted (500 eggs in 50 g of diet for each replicate, 30 replicates for each diet) and following pupation the number of successfully developed pupae was used to estimate egg-pupa recovery. Ten pupae per replicate were individually weighted after 6 days of pupation to estimate average pupal weight. The percentage of emergence per replicate was estimated from the produced pupae. Adults for metal estimations were freeze-dried, and their wet and dry weight recorded. For the determination of lipid content, 30 male and 30 female newly emerged individuals per dietary treatment were collected and stored individually at −20 °C until chemical analysis. A protocol using digestion with sulfuric acid and vanillin reagent (Warburg and Yuval 1996) was used to extract lipids from individual flies, and the standard curve was constructed using incremental concentrations of Triolein (Sigma, St. Louis, MO) as the lipid standard. The lipid determination was performed by reading the vanillin-reaction in 96-well microplates at 490 nm in an ELISA-reader (Nestel et al. 2004). For soluble protein quantification, a different set of individuals (10 males and 10 females) per dietary scheme were used. Flies were individually homogenized in PBS, and protein development was conducted using the Bradford reagent (Bio-Rad laboratories, Richmond, CA). Protein content per fly was determined by reading at 595 nm, using bovine serum albumin (Sigma, St. Louis, MO) as a standard (Nestel et al. 2004).

### Statistical analysis

All metal content data (species-replications) were subjected to one-way analysis of variance (ANOVA), followed by Tukey’s HSD post hoc pairwise comparisons. Ordination and classification of specific metal-contents in the Tephritidae and Drosophilidae species was inferred using the average metal content per species in a principal component analysis. The first principal component was further employed to classify species in a dendrogram system. All statistical inferences were conducted with the software JMP^®^ for Windows (SAS).

## Results

### Metal content in recently established laboratory populations and long-established laboratory cultures of the same species

For each of the species, *B. oleae*, *B. dorsalis* and *C. capitata*, a recently established colony and additional populations of a different origin but long established in the laboratory (at least 40 years for Mediterranean fruit fly and olive fly colonies and 5 years for *B. dorsalis*) were compared (Table 1). The latter were assumed to have undergone significant bottleneck selection. Accumulation of iron, zinc, copper and manganese during larval and pupal development was taken to be the content at adult emergence (Table 2). A one-way ANOVA revealed significant differences between species and populations. *C. capitata* showed significantly higher iron than the two *Bactrocera* species. Moreover, the Israeli population of *C. capitata* showed significantly higher levels of zinc and copper too, possibly related to the slightly modified diet on which they were cultivated (footnote to Table 2 and “Materials and methods” section above) or to genetic background, but otherwise little differences were observed between populations of the same species, whether colonies were recently caught or longstanding. Similar patterns and lack of differences between populations of the same species were already described for various *Drosophila* species and strains (Sadraie and Missirlis 2011).

**Table 2:** Metal content (mg metal/g dry weight) of recently eclosed adult flies presented as averages ± standard deviation with number of independent biological replicates indicated as N

Species/Origin	Diet	N	Fe (mg/g)	Zn (mg/g)	Cu (mg/g)	Mn (mg/g)
*Bactrocera oleae*						
1-1 Italy—wild	i	7	0.13 ± 0.03^cd^	0.13 ± 0.02^bc^	0.008 ± 0.003^b^	0.008 ± 0.001^b^ *
1-2 Greece—lab	i	8	0.14 ± 0.05^cd^	0.12 ± 0.03^bc^	0.010 ± 0.003^b^	0.010 ± 0.001^ab^ *
1-3 Israel—lab	i	7	0.11 ± 0.03^d^	0.11 ± 0.02^c^	0.009 ± 0.004^b^	0.006 ± 0.000^b^ *
*Bactrocera dorsalis*						
2-1 Thailand—wild	ii	9	0.19 ± 0.03^cd^	0.19 ± 0.04^b^	0.008 ± 0.002^b^	0.041 ± 0.025^ab^
2-2 China—lab	ii	9	0.19 ± 0.05^cd^	0.18 ± 0.06^bc^	0.008 ± 0.002^b^	0.025 ± 0.006^ab^**
2-3 Hawai—lab	ii	9	0.18 ± 0.08^cd^	0.18 ± 0.04^bc^	0.008 ± 0.004^b^	0.051 ± 0.041^ab^
*Ceratitis capitata*						
13-1 Argentina—wild	iii	9	0.24 ± 0.09^bc^	0.17 ± 0.04^bc^	0.006 ± 0.002^b^	0.028 ± 0.016^ab^
13-2 Austria—lab	iii	9	0.30 ± 0.13^ab^	0.18 ± 0.05^bc^	0.010 ± 0.004^b^	0.054 ± 0.016^a^
13-3 Israel—lab	iii***	7	0.41 ± 0.04^a^	0.27 ± 0.03^a^	0.028 ± 0.003^a^	0.041 ± 0.006^ab^
Statistics****						
1-way ANOVA F ratio	F_(8,65)_		12.8, *p* < 0.001	10.3, *p* < 0.001	35.1, *p* < 0.001	3.7, *p* < 0.002*****

Different populations, wild and lab-adapted, from three representative species are shown

* Average of three determinations shown because in other samples (Mn) was below the detection limit

** Average of six determinations shown because in other samples (Mn) was below the detection limit

*** This diet contained different preservatives

(HCl 1.6 % and Nipagin 0.4 % instead of Sodium Benzoate 0.5 % and Citric Acid 1.8 %)

**** Within columns, averages followed by different letters [a, b, c, d] indicate statistically significant differences in pair-wise comparisons using the Tukey HSD test and *p* < 0.05

***** F_(8,50)_

### Metal content in species of Tephritidae

Eleven additional species of Tephritidae and a wild type *D. melanogaster* as control were added to the analysis (Table 3). Fruit fly species differed significantly in their metal content (*p* < 0.001). *Bactrocera oleae* showed a low concentration of all four metals compared to the averages of all other species tested. In contrast, *C. capitata* metal contents were generally in the upper part of the range. The high degree of zinc accumulation in *A. grandis* was notable; a finding unlikely to be attributable to the zucchini diet of this oligophagous species, as *D. ciliatus* larvae were also cultivated on zucchini but did not accumulate zinc to a similar extent (Table 3). *Bactrocera papayae* was found to accumulate more manganese compared to all other species, and *B. oleae* was strikingly manganese-poor. In more than half of the *B. oleae* samples manganese was undetectable and not even included in the ANOVA.

**Table 3:** Metal content (mg metal/g dry weight) of Tephritidae species

Species	Diet	N	Fe (mg/g)	Zn (mg/g)	Cu (mg/g)	Mn (mg/g)
1. *Bactrocera oleae*	i	22	0.13 ± 0.04^c^	0.12 ± 0.03^c^	0.009 ± 0.003^bc^	0.008 ± 0.002^d^
2. *Bactrocera dorsalis*	ii	27	0.18 ± 0.04^bc^	0.18 ± 0.05^b^	0.008 ± 0.003^c^	0.040 ± 0.021^c^
3. *Bactrocera tryoni*	ii	9	0.18 ± 0.04^bc^	0.22 ± 0.03^b^	0.010 ± 0.002^abc^	0.038 ± 0.013^cd^
4. *Bactrocera cucurbitae*	ii	9	0.15 ± 0.05^bc^	0.21 ± 0.05^b^	0.010 ± 0.002^abc^	0.037 ± 0.017^cd^
5. *Bactrocera carambolae*	ii	9	0.23 ± 0.05^ab^	0.21 ± 0.05^b^	0.009 ± 0.002^abc^	0.044 ± 0.016^bcd^
6. *Bactrocera papayae*	ii	9	0.24 ± 0.05^ab^	0.23 ± 0.05^b^	0.010 ± 0.005^abc^	0.100 ± 0.048^a^
7. *Bactrocera invadens*	ii	9	0.19 ± 0.03^bc^	0.18 ± 0.02^bc^	0.006 ± 0.003^c^	0.078 ± 0.043^ab^
8. *Bactrocera zonata*	ii	9	0.20 ± 0.07^bc^	0.22 ± 0.08^b^	0.006 ± 0.003^c^	0.049 ± 0.028^bc^
9. *Bactrocera philippinensis*	ii	9	0.23 ± 0.06^ab^	0.20 ± 0.05^b^	0.011 ± 0.004^abc^	0.024 ± 0.006 ^cd^
10. *Anastrepha ludens*	ii	9	0.21 ± 0.05^bc^	0.24 ± 0.04^b^	0.017 ± 0.004^a^	0.048 ± 0.015^bc^
11. *Anastrepha fraterculus*	ii	9	0.19 ± 0.04^bc^	0.22 ± 0.06^b^	0.010 ± 0.002^abc^	0.039 ± 0.026^cd^
12. *Anastrepha grandis*	iv	8	0.17 ± 0.05^bc^	0.34 ± 0.09^a^	0.012 ± 0.004^abc^	0.028 ± 0.011^cd^
13. *Ceratitis capitata*	iii	25	0.31 ± 0.12^a^	0.20 ± 0.06^b^	0.014 ± 0.010^a^	0.041 ± 0.018^c^
14. *Dacus ciliatus*	iv	7	0.17 ± 0.03^bc^	0.23 ± 0.04^b^	0.016 ± 0.005^a^	0.011 ± 0.002^cd^
15. *Drosophila melanogaster*	v	9	0.19 ± 0.03^bc^	0.23 ± 0.07^b^	0.015 ± 0.003^abc^	0.017 ± 0.008^cd^
Statistics*						
1-way ANOVA F ratio	F_(14,164)_		8.5, *p* < 0.001	9.8, *p* < 0.001	5.5, *p* < 0.001	9.2, *p* < 0.001**

* Within columns, averages followed by different letters [a, b, c, d] indicate statistically significant differences in pair-wise comparisons using the Tukey HSD test and *p* < 0.05

** F_(8,149)_

### Trends in metal accumulation in genera of Tephritidae

Species were pooled together by genus of Tephritidae to detect patterns of metal accumulation at this systematic level (Table 4). Four genera were compared: the *Bactrocera* were represented by nine species, the *Anastrepha* by three, whereas only one representative was available for *Ceratitis* and *Dacus.* Differences between genera in metal accumulation were significant for the four metals tested (*p* < 0.001 for iron, zinc and copper, *p* < 0.05 for manganese): the *Bactrocera* showed low concentration of copper, the *Anastrepha* accumulated high levels of zinc and *Ceratitis* accumulated high levels of iron. Although these differences were statistically significant, they still amounted to less than a two-fold variation. Only manganese content in *D. ciliatus* varied by a factor greater than two, but as manganese concentrations showed high variability (an observation also made in the Drosophilidae; Sadraie and Missirlis 2011) the probability of this finding being the effect of chance remains considerable. More representative species from the *Dacus* and *Ceratitis* genera need to be tested before our observations can be generalized.

**Table 4:** Metal content (mg metal/g dry weight) per genus of Tephritidae and comparison with the Drosophilidae family

Species/Genus/Family	N	Fe (mg/g)	Zn (mg/g)	Cu (mg/g)	Mn (mg/g)
*Bactrocera* sp.	112	0.18 ± 0.06^b^	0.18 ± 0.05^b^	0.009 ± 0.003^b^	0.045 ± 0.035^a^
*Anastrepha* sp.	26	0.19 ± 0.05^b^	0.26 ± 0.09^a^	0.013 ± 0.004^a^	0.039 ± 0.020^ab^
*Ceratitis capitata*	25	0.31 ± 0.12^a^	0.20 ± 0.06^b^	0.014 ± 0.010^a^	0.041 ± 0.018^ab^
*Dacus ciliatus*	7	0.17 ± 0.03^b^	0.23 ± 0.04^ab^	0.016 ± 0.005^a^	0.011 ± 0.002^b^
Statistics^*^					
1-way ANOVA F ratio	F_(3,166)_	22.7, *p* < 0.001	12.4, *p* < 0.001	14.6, *p* < 0.001	3.3, *p* < 0.05**
Average Tephritidae	14	0.20 ± 0.04	0.21 ± 0.05	0.011 ± 0.003	0.042 ± 0.024
Average Drosophilidae***	9	0.22 ± 0.07	0.18 ± 0.07	0.018 ± 0.006	0.028 ± 0.013

^*^Within columns, averages followed by different letters [a, b, c, d] indicate statistically significant differences in pair-wise comparisons using the Tukey HSD test and *p* < 0.05

^**^F_(3,151)_

^***^Data from nine *Drosophila* species taken from (Sadraie and Missirlis 2011)

### Conserved metal accumulation in two Diptera families

**Fig. 1:**
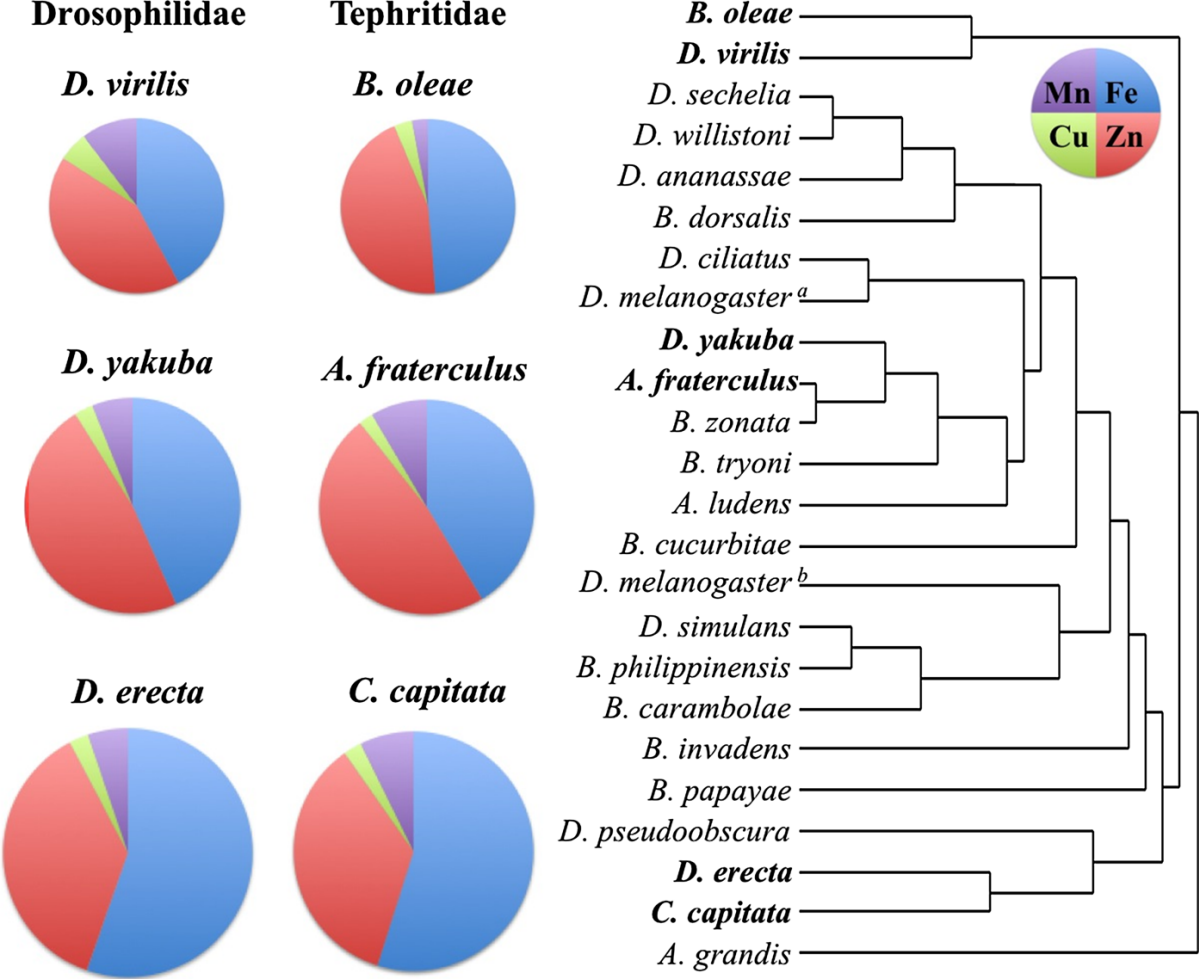
Cluster analysis based on the average values of metal composition of 23 Dipteran species belonging to Tephritidae and Drosophilidae families. Clustering by 1st principal component. The relative distributions of iron (*blue*), zinc (*red*), manganese (*purple*) and copper (*green*) of three representative species that cluster together are depicted on the *left*, with circle area scaled to total metal content (per g dry weight). For numerical values see Table 3 for the Tephritidae and (Sadraie and Missirlis 2011) for the Drosophilidae. *Drosophila melanogaster* appears in both references and was raised in different diets between the two studies. It is notable that Tephritidae outliers *B. oleae* and *A. grandis* have a very narrow host range and were raised on different diets

A principal component analysis was carried out using the average values for each metal, including the values previously determined in nine wild-type *Drosophilidae* species (Sadraie and Missirlis 2011). Cluster analysis is presented in a dendrogram form (Fig. 1). The results grouping together Drosophilidae and Tephritidae species suggest that relative metal content is a poor predictor for evolutionary relatedness between fly species and families of flies (Han and Ro 2009; Stratikopoulos et al. 2009). As an example, *Drosophila virilis* and *Drosophila erecta*, previously found to accumulate iron and other metals differentially compared to the other Drosophilidae, grouped very closely with *B. oleae* and *C. capitata*, respectively. Depicting graphically the relative metal content of representative species demonstrates remarkably similar patterns between all species regardless of their statistical clustering. *Drosophila yakuba* and *Anastrepha* *fraterculus* and the outliers already mentioned were chosen as examples to illustrate this point (Fig. 1).

The average metal composition was calculated for all fourteen species included in this study and compared with the average metal composition of nine species of Drosophilidae (Sadraie and Missirlis 2011). Despite the differences in food composition between the species belonging to these different families, they showed similar iron, zinc or manganese concentrations and only a small difference could be detected for copper, which accumulated at a lower level in the Tephritidae (Table 4). Thus, the pattern that emerged from the comparisons between the two families of Diptera suggests that metal homeostasis is well conserved between these insect families.

### Metal accumulation in *C. capitata* cultivated on different diets

**Fig. 2:**
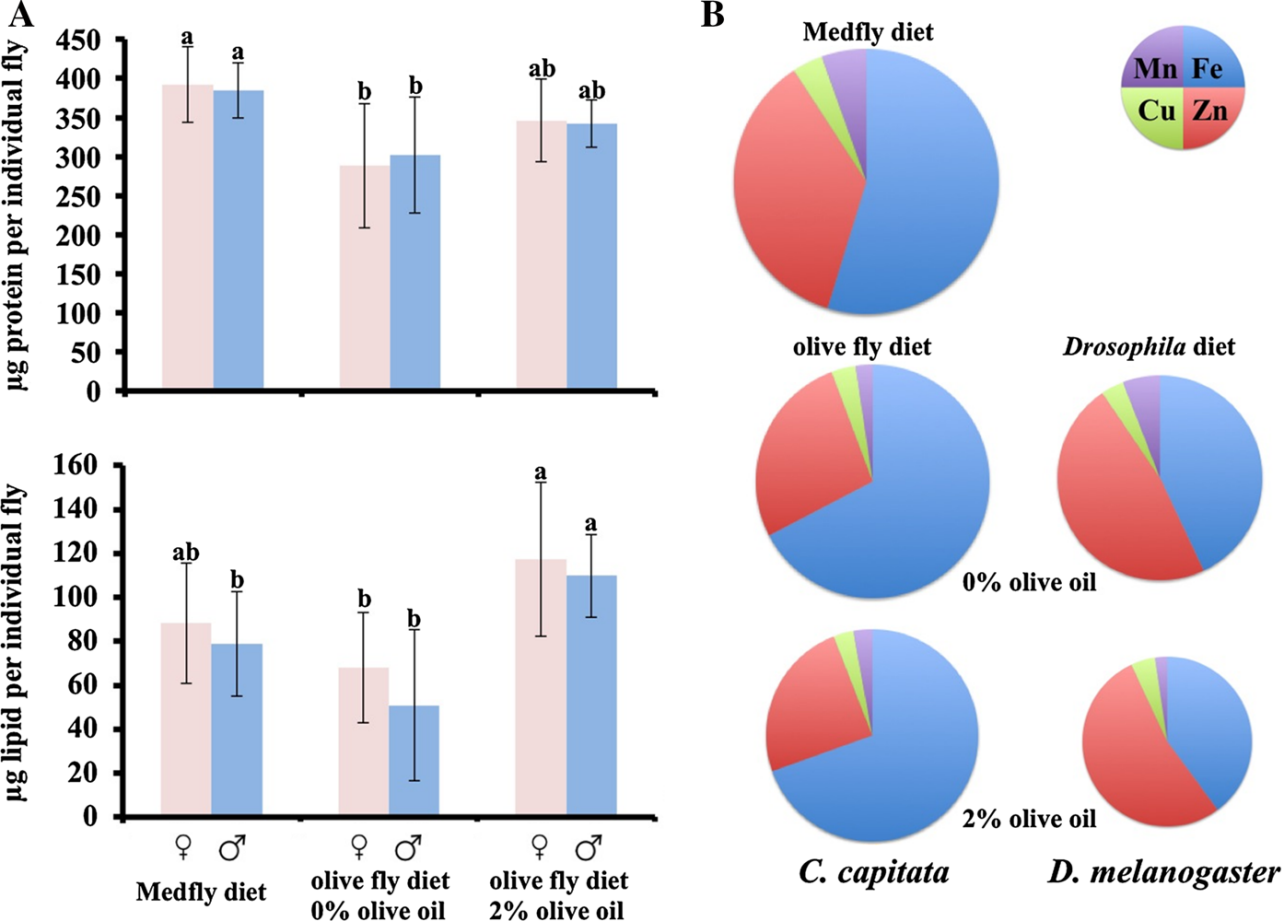
Effect of three different dietary schemes on *C. capitata* protein, lipid and metal content. **a** A population of *C. capitata* cultivated in Israel (13–3) was raised on the diet of *B. oleae* (i—2 % olive oil) and also on a similar diet but omitting the olive oil (i—0 %). Soluble protein and lipid content (higher and lower graphs, respectively) was determined for thirty individual male flies and thirty females per dietary treatment. Mediterranean fruit fly protein content was significantly lower when the flies were raised on 0 % olive oil (F_2,27_ ratio = 6.62; *p* = 0.004 for males, F_2,27_ ratio = 7.02; *p* = 0.003 for females), and lipid content was significantly higher when they were raised on olive fly diet containing 2 % olive oil (F_2,26_ ratio = 10.95; *p* = 0.0004 for males, F_2,30_ ratio = 8.08; *p* = 0.001 for females). **b** The relative distribution of iron (*blue*), zinc (*red*), manganese (*purple*) and copper (*green*) of the *C. capitata* on the three different diets is depicted on the left, with circle area scaled to total metal content (for values see Table 5). A single experiment with *D. melanogaster* is also shown for comparison. Olive oil leads to a reduction of metal content per mg dry weight in both species tested

The possible effect of olive oil on metal accumulation was tested in a preliminary experiment by cultivating *D. melanogaster* in its normal diet supplemented with 2 % olive oil. It was found that the addition of olive oil to the diet of this species resulted in significant lethality with fewer than 10 % of larvae initiating metamorphosis to become fully developed pupae. The few adults able to emerge from this treatment were also short-lived. As a result, a single sample was obtained for our metal determination assay. In contrast to *D. melanogaster*, the polyphagous *C. capitata* thrived on the *B. oleae* diet, with 48 % of the embryos reaching the pupal stage on the *C. capitata* normal diet and 30 % when cultivated on the *B. oleae* diet. Well over 90 % of these pupae successfully emerged in both conditions and gave rise to adults of similar body weight. Total protein and lipid contents were also determined per individual, controlling for gender (Fig. 2). Only minor differences in protein and lipid content were contrasted by a dramatic decrease for zinc, copper and manganese (Table 5). The zinc and manganese concentration were well below the Tephritidae average. Iron was also reduced, though only mildly, remaining at a relatively high value. Finally, *C. capitata* was compared with or without olive oil. The addition of olive oil further diminished zinc and copper accumulation and also caused a detectable reduction in iron accumulation. Therefore, it appears that olive oil has metal chelating activity, at least as indirectly judged by its effect on metal accumulation when fed to insects (Fig. 2 and Table 5).

**Table 5:** Effect of switching *Ceratitis capitata* on the diet of *Bactrocera oleae* on metal content

Species	Diet*	N	Fe (mg/g)	Zn (mg/g)	Cu (mg/g)	Mn (mg/g)
3-3 *Ceratitis capitata*	iii—0 %	7	0.41 ± 0.04^a^	0.27 ± 0.03^a^	0.028 ± 0.003^a^	0.041 ± 0.006^a^
3-3 *Ceratitis capitata*	i—2 %	7	0.34 ± 0.03^b^	0.12 ± 0.02^c^	0.015 ± 0.002^c^	0.014 ± 0.003^b^
3-3 *Ceratitis capitata*	i—0 %	6	0.40 ± 0.04^a^	0.16 ± 0.03^b^	0.020 ± 0.003^b^	0.014 ± 0.003^b^
Statistics**						
1-way ANOVA F ratio	F_(2,17)_		7.7, *p* < 0.005	54.5, *p* < 0.001	42.4, *p* < 0.001	101.6, *p* < 0.001
*Drosophila melanogaster*	v—2 %	1	0.12	0.16	0.014	0.007
*Drosophila melanogaster*	v—0 %	1	0.19	0.21	0.016	0.026

* Olive oil inclusion as indicated

** Within columns, averages followed by different letters [a, b, c, d] indicate statistically significant differences in pair-wise comparisons using the Tukey HSD test and *p* < 0.05

## Discussion

### Metal homeostasis in Drosophilidae and Tephritidae

In nine species of Drosophilidae iron and zinc concentrations were about 200 µg/g of the adult dry weight, and manganese and copper to about one tenth of this level (Sadraie and Missirlis 2011). A similar result holds in the present study for 14 species of Tephritidae, suggesting a conservative selection mechanism has been in place in natural populations over a period of approximately 100 million years (Beverley and Wilson 1984; Wulbeck and Simpson 2000). An alternative explanation is that the fruit fly genomic mutations do not normally affect the metallome, however mutations affecting metal accumulation are known in laboratory strains of *D. melanogaster* (for example reducing iron, Bettedi et al. 2011; reducing copper, Hua et al. 2011; reducing zinc, Afshar et al. 2013; reducing manganese, Freeman et al. 2013), which in general are non-lethal and show little effect on fitness.

### Effect of diet on metal accumulation

An established way to alter metal content of a given stain of *D. melanogaster* is to feed the larvae a diet enriched with a metal salt (Bettedi et al. 2011; Bonilla-Ramirez et al. 2011; Georgieva et al. 1999; Mora et al. 2014) or a metal chelator (Gutierrez et al. 2010; Sanokawa-Akakura et al. 2010; Singh et al. 2013; Soriano et al. 2013; Yang et al. 2005). This manipulation readily alters the levels of metal accumulated in the adult flies. Therefore, it was expected that flies raised on different diets might as a consequence show changes in their metal homeostasis unrelated to their different genetic background. The two species with the most striking differences in this study were *B. oleae*, which had low levels of metals tested and *C. capitata*, which had high levels. These two species are also opposites with respect to their range of natural hosts. *B. oleae* is strictly monophagous feeding only on the olive fruit mesocarp (Tzanakakis 2003), whereas *C. capitata* feeds on hundreds of fruits or vegetables worldwide (Liquido et al. 1991; Hancock et al. 2000; De Meyer et al. 2002). This allowed testing the effect of cultivating *C. capitata* on a specialized *B. oleae* diet, but not vice versa. The result of this experiment showed that the diet is a parameter that cannot be ignored in metal homeostasis studies as *C. capitata* showed a two-fold reduction in zinc and copper and a three-fold reduction in manganese when cultivated on a *B. oleae* diet (Table 5). The effect on iron accumulation was minimal, giving rise to two further considerations. On the one hand, it may be important to test the sustainability of *C. capitata* on this diet for a few generations before reassessing its impact on iron homeostasis. A strong maternal contribution of ferritin iron has been reported in *D. melanogaster* (Gonzáles-Morales et al. 2014), which raises the question on the conservation of a similar mechanism in *C. capitata* resulting in a delay of any dietary effects on metal chelation. On the other hand, when grouping the insects according to their natural range of hosts and looking for any correlating change in any of the four metals, a clear series for iron content (as µg/g dry weight) could be detected, with monophagus *B. oleae* (0.13) < oligophagus (see Table 1) flies (0.17) < polyphagus flies (0.21) < *C. capitata* (0.31). At present, we do not know if there is any causal reason for this correlation or whether it has ecological relevance.

### Metal chelating properties of olive oil

Olive oil constitutes a central ingredient of the human diet in the Mediterranean countries and a large number of studies have been carried out on its nutritional properties in humans (reviewed in Boskou 2006; Cicerale et al. 2012; Covas et al. 2009). However, studies on its metal chelating properties when fed to animals are completely lacking. When we first noted that *B. oleae* was manganese-deficient and showed low iron and zinc accumulation compared to other *Bactrocera* species, we wondered whether this could be due to the addition of olive oil to its diet. Our attempt to address this question in *D. melanogaster* was hindered with a surprising high mortality at all stages of the life cycle. We were able to collect enough survivors for one comparative experiment (with flies cultivated on a normal diet in parallel), the results of which showed a drop in all metals in the*Drosophila * flies fed with olive oil (Table 5). The experiment with the Tephritid *C. capitata* could be repeated a number of times and showed a statistically significant drop in iron, copper and zinc but not manganese (although it should be noted that some other component in the *B. oleae* diet may be acting as a strong manganese chelator). *Ceratitis capitata* has been broadly used as a model for nutritional studies (Chang et al. 2000; Nash and Chapman 2014; Nestel et al. 2004, 2005; Papanastasiou et al. 2013) and we suggest that it could also become a good model system to investigate hypotheses regarding the metabolism and nutrition of metals.

### Storage of metals versus co-factor requirements

Our results showed a very similar overall pattern of metal accumulation in Drosophilidae and Tephritidae in spite of cultivating these flies on different media. A working hypothesis to explain the metal conservation is that transition metals are required primarily as co-factors on vital metabolic enzymes and zinc for transcription factors, metalloproteases, carbonic anhydrase. Thus, the metallation of the proteins carrying out the insect’s physiological requirements is reflected in the relative stable accumulation pattern of metals across the two Diptera families. When iron availability is manipulated in the diet, there is a corresponding change in the iron storage protein ferritin (Georgieva et al. 1999; Jiang et al. 2014; Missirlis et al. 2006, 2007; Tang and Zhou 2013), which would be the second—and more flexible—way to change insect metal accumulation without affecting its physiology. However, apart from insect ferritins (Pham and Winzerling 2010), we know very little about metal storage in the case of the other metals (Gutierrez et al. 2013). Excess copper, for example, frequently used as an insecticide, directly affects insect immunity (Polkki et al. 2012) and readily accumulates in flies (Bettedi et al. 2011), possibly bound to metallothioneins (McNulty et al. 2001; Egli et al. 2006). Some variability in metal accumulation can be attributed to a change in storage capacity, as seen in the differential ferritin accumulation in *D. virilis* and *D. erecta* (Sadraie and Missirlis 2011). However, it is at present difficult to study the same question for the other metals in the absence of concrete knowledge of their storage systems. A further complication could arise from the fact that insects appear to use different metalloenzymes during their different stages of development (Marelja et al. 2014), calling for metal storage mechanisms to remain operational also during developmental progression (Gonzáles-Morales et al. 2014; Tang and Zhou 2013). The small size of insects makes a tissue-by-tissue analysis difficult, yet tissue specificity of metal accumulation (Mehta et al. 2009) would also be informative regarding the internal mobilization and use of the metal ions. The role of the intestinal microbiota in metal absorption (Ben-Yosef et al. 2010; Erkosar Combe et al. 2014; Hobbie et al. 2012) and the role of symbionts, such as *Wolbachia*, that interact with the host’s metabolism (Kosmidis et al. 2014; Kremer et al. 2009) should also be considered. Finally, hematophagous insects are bound to have developed specialized strategies against metals because of their specialized diet (Ribeiro et al. 2014; Zhou et al. 2007). Nevertheless and despite of all the variables that remain to be tested, the major conclusion of the study was that metal homeostasis is conserved in many species of fruit flies.
